# The role played by ailanthone in inhibiting bone metastasis of breast cancer by regulating tumor-bone microenvironment through the RANKL-dependent pathway

**DOI:** 10.3389/fphar.2022.1081978

**Published:** 2023-01-05

**Authors:** Yajun Wang, Zeyuan Zhong, Miao Ma, Yannan Zhao, Chongjing Zhang, Zhi Qian, Biyun Wang

**Affiliations:** ^1^ Department of Breast Cancer and Urological Medical Oncology, Fudan University Shanghai Cancer Center, Shanghai, China; ^2^ Department of Oncology, Shanghai Medical College, Fudan University, Shanghai, China; ^3^ Shanghai Medical College, Fudan University, Shanghai, China; ^4^ Department of Orthopedics, Shanghai Pudong Hospital, Fudan University Pudong Medical Center, Shanghai, China; ^5^ Department of Orthopedics, Lanzhou University Second Hospital, Lanzhou, China; ^6^ The Second Clinical Medical College, Lanzhou University, Lanzhou, China; ^7^ Institution of Orthopedic Diseases, Zhangye People’s Hospital Affiliated to Hexi University, Zhangye, China

**Keywords:** breast cancer, ailanthone, bone metastasis, osteoclast, MDA-MB-231

## Abstract

**Introduction:** Bone metastasis of breast cancer (BC) is a process in which the disruption of the bone homeostatic microenvironment leads to an increase in osteoclast differentiation. *Ailanthus altissima* shows an inhibitory effect on osteoclast differentiation. Ailanthone (AIL) refers to a natural compound isolated from *Ailanthus altissima*, a Chinese herbal medicine, and has effective anti-tumor activity in numerous cell lines. Its impact on bone metastases for BC is yet unclear.

**Methods:** We measured the effect of AIL on MDA-MB-231 cells by wound healing experiments, Transwell and colony formation experiment. Using the Tartrate-resistant Acid Phosphatase (TRAP) staining tests, filamentous (F-actin) staining and bone resorption test to detect the effect of AIL on the osteoclast cell differentiation of the Bone Marrow-derived Macrophages (BMMs), activated by the MDA-MB-231 cell Conditioned Medium (MDA-MB-231 CM) and the Receptor Activator of Nuclear factor-κB Ligand (RANKL),and to explore its possibility Mechanisms. *In vivo* experiments verified the effect of AIL on bone destruction in breast cancer bone metastasis model mice.

**Results:** *In vitro*, AIL significantly decrease the proliferation, migration and infiltration abilities of MDA-MB-231 cells at a safe concentration, and also reduced the expression of genes and proteins involved in osteoclast formation in MDA-MB-231 cells. Osteoclast cell differentiation of the BMMs, activated by MDA-MB-231 CM and RANKL, were suppressed by AIL in the concentration-dependent manner. Additionally, it inhibits osteoclast-specific gene and protein expression. It was noted that AIL inhibited the expression of the osteoclast differentiation-related cytokines RANKL and interleukin-1β (IL-1β) that were secreted by the MDA-MB-231 cells after upregulating the Forkhead box protein 3 (FOXP3) expression. Furthermore, AIL also inhibits the expression of the Mitogen-Activated Protein Kinase (MAPK), Phosphoinositide 3-kinase (PI3K)/protein kinase B (AKT), and Nuclear factor-κB Ligand (NF-κB) signaling pathways, which then suppresses the MDA-MB-231CM-induced development of Osteoclasts.

**Conclusion:** Our study shows that AIL blocks osteoclast differentiation in the bone metastasis microenvironment by inhibiting cytokines secreted by BC cells, which may be a potential agent for the treatment of BC and its secondary bone metastasis.

## Introduction 

Breast Cancer (BC) is regarded as a most prevalent form of cancer that affects women across the globe, wherein 90% of cancer-linked deaths are due to tumor metastasis (Liang et al., 2020). Non-metastatic BC patients show a 5 year Overall Survival (OS) duration of >80%; however, the occurrence of distant metastasis can significantly reduce the rate to about 26% (Yardley, 2016). Because of the high vascularization of bone trabecula, the BC cells were successfully dispersed in blood, while the bone was regarded as the preferred site for metastasis (Theriault and Theriault, 2012). In the past few years, development of cancer treatment has significantly improved the OS rate of BC patients; however, the none metastasis risk has also increased (Nakai et al., 2019). Bone metastasis accounts for about 75% of BC metastatic cases (Liang et al., 2020), and the 5 year OS rate was only 22.8% (Xiong et al., 2018). Triple negative breast cancer (TNBC) is highly aggressive and has a strong ability of distant metastasis. It is only sensitive to chemotherapy, with relatively limited treatment options and poor prognosis. And it is osteotropic and prone to bone metastasis (Zhang et al., 2020). Bone metastasis can cause severe bone pain and increase the occurrence of bone Related Events (SRE), like radiotherapy, spinal cord compression, pathological fracture, and palliative bone surgery (Hatoum et al., 2008; Jakob et al., 2022). Bone metastasis, pain, and SRE not only increase the medical cost and death risk but also greatly affect the Quality of Life (QoL) of BC patients (Koizumi et al., 2010; Cetin et al., 2014).

Bone metastasis caused by BC is mainly osteolytic lesions, which are closely associated with the osteophagocytic process of osteoclasts (Guise et al., 2006). BC cells can enhance the differentiation of osteoclasts, destroy normal bone homeostasis, and form a vicious circle (Chen et al., 2010). Currently, bone metastasis in BC mainly involves seven signaling pathways, including OPG/RANK/RANKL, MAPK-ERK-cFOS, PI3K-AKT-mTOR, and WNT signaling pathways (Song et al., 2022). On osteoclast precursors, RANKL and homologous receptor RANK (receptor activator of RANK, NF-κB), activate a variety of intracellular signal pathways and initiate osteoclast cell differentiation and maturation by stimulating the transcription and expression of the osteoclast-linked genes like TRAP, c-Fos, Cathepsin K (CTSK), and NFATc1 (Mcdonald et al., 2021). Osteoprotegerin (OPG) is an endogenous bait receptor of RANKL, which can inhibit the generation of osteoclasts (Teitelbaum and Ross, 2003). By modulating the RANKL to OPG ratio, the cytokines like PTH/PTHrP, IL-1, IL-6, and IL-11, indirectly or directly secreted by the tumor cells, enhance the osteoclast differentiation and maturation, which facilitates the process of osteoclast bone absorption (Weilbaecher et al., 2011). It is essential to prevent growth and development of tumors and the osteoclast-induced osteolysis process in BC patients, from the clinical perspective (Zou et al., 2022). Therefore, it is a feasible way to prevent and treat bone metastasis of breast cancer by inhibiting some cytokines and RANKL secreted by tumor cells, thereby inhibiting their activated downstream signal pathways.

Several clinically-important antineoplastic drugs have been obtained from plants and herbal extracts, wherein a few of these drugs can significantly prolong the OS duration of BC patients. *A. altissima* inhibits RANKL-induced osteoclast differentiation (Youn et al., 2008). AIL is a pentacyclic diterpene lactone Compound, extracted from *A. altissima*, which displays anti-inflammatory, anti-tumor, anti-malaria, and other effects (He et al., 2016; Ding et al., 2020). It has been proved that AIL inhibits the PI3K/AKT signaling pathway by lowering the PI3K and AKT phosphorylation in many tumors, thereby playing an anti-tumor role (Ding et al., 2020). AIL exerts its anti-osteosarcoma effect by inhibiting PI3K/AKT pathway in MG63 cells (Kong et al., 2019). AIL blocks the mitogen-activated protein kinase (MAPK) and mTOR signaling pathways in the Schwann cells in a miR-21-dependent manner (Yang et al., 2018). Another study showed that AIL significantly blocked the proliferation of human BC cells and activated their apoptosis in the concentration-dependent manner (Wang et al., 2018). The above research highlights the potential application of AIL in treating BC patients. However, there is currently no information available about the treatment or mechanism used by AIL for BC bone metastases. This study was conducted to evaluate the impact of AIL on BC-led bone damage and determine its potential molecular mechanisms.

## Materials and methods

### Reagents and antibodies used in this study

AIL (>98% purity) was procured from MedChemExpress Ltd. (NJ, United States), and dissolved using the Phosphate Buffered Saline (PBS) to formulate a stock solution of AIL (10 mmol/L), which was then stored at the temperature of −20°C till further use. It was then subsequently diluted using the sterile cell culture medium or PBS, respectively, to conduct the cell and animal experiments. ABclonal Technology (Wuhan, China) supplied the primary antibodies for CXCR4, MMP9, TGF-β, FOXP3, β-actin, and PTHrP. Cell Signaling Technologies (Beverly, United States) provided the primary antibodies for DCSTAMP, CTSK, TRAP, AKT, P-AKT, Vinculin, JNK, P65, P-P65, NFATc1, P38, P-P38, ERK, P-ERK, P-JNK, IκBα, P-IκBα, PI3K, and P-PI3K. The RANKL and IL-1β ELISA kits were procured from LinkedIn Biology (Zhejiang, China). The CCK-8 test kit was also obtained from Dojindo (Japan). The leukocyte acid phosphatase staining kit was also purchased (Sigma Aldrich Ltd., United States). R&D Systems (Minnesota, United States) supplied the recombinant m-RANKL and M-CSF molecules. Other chemicals such as the penicillin-streptomycin antibiotic solution, high sugar DMEM medium, alpha-modified Minimum Essential Medium (α-MEM), and Fetal Bovine Serum (FBS) were obtained from Thermo Fisher Scientific Ltd. (Scoresby, Australia).

### Cell culture

The femur of C57BL/6 mice (aged 6 weeks) serves as the source of Bone Marrow-derived Macrophages (BMMs). The BM cavity was rinsed with PBS, and the solution was centrifuged. The centrifuged cell pellet was cultured on the α-MEM medium that contained 10% FBS, and supplemented with the penicillin and streptomycin double antibiotic solution (1% v/v). The BMM cells were cultivated at 37°C for 24 h under conditions of 5% CO_2_. The supernatant and suspension cells were transferred to a fresh 10 cm culture plate, where every well contained the α-MEM medium, 10% FBS, double antibiotic solution (1% v/v), and M-CSF (20 ng/ml). Fresh medium was added to the wells after 2 days and cells were allowed to cultivate. This study used 2–3 generations of cells, with a confluence of >80%. MDA-MB-231 cells were cultivated in the high-glucose DMEM medium, supplemented with FBS (10% v/v) and a double antibiotic (1% v/v) solution, at 37°C and 5% CO_2_. The old medium was pipetted out and fresh medium was added to the wells after 2 days. The cells were subcultured when the confluence level exceeded 80%.

### Construction of MDA-MB-231-Luc cells

The MDA-MB-231 cells were cultivated in a 10-cm culture dish, where the cells acquired a 20%–30% confluence level. The cell culture medium (4 ml) with retrovirus was collected and mixed with polybrene (40 µl of 10 mg/ml solution), filtered with a filter membrane (0.45 µm), and mixed with the cell suspension. The solution was mixed thoroughly and incubated at 37°C, overnight. Then, the medium containing retrovirus was pipetted out and the complete culture medium (10 ml) was added. Thereafter, the cells were normally cultured at 37°C for 48 h, collected and cultured with hygromycin B for 14 days and the positive cells were selected.

### Cell viability analysis

Cell viability was assessed using the manufacturer’s instructions for the CCK-8 (Cell Count Kit-8). The MDA-MB-231 cells were inoculated into the 96-well culture plate (1 × 10^4^ wells), incubated at 37°C with different AIL concentrations for 48 h, and were rinsed using PBS. The serum-free culture medium (100 μl) containing CCK-8 solution (10 μl) was added to every well, and cell culture plates were incubated for 60 s, at 37°C with 5% CO_2_. Finally, the optical density of the cell suspension was assessed at 450 nm, using a microplate reader. The activity of BMM cells was also determined with the help of the above technique.

### Wound healing experiments

The MDA-MB-231 cells were digested and inoculated in the 6-well culture plate. Each well contained 5 × 10^5^ cells. The culture plate was incubated overnight, and a gun head (200 µl) was used to draw a 0.5 cm wide line at the base of every well. The medium was changed and fresh DMEM with varying concentrations of AIL (0, 0.625, and 1.25 μmol/L) were added to different wells, and the plate was incubated for either 24 h or 48 h, respectively. These cells were fixed using paraformaldehyde (4%), stained using gentian violet dye, dried, and observed under the optical microscope (Nikon, Japan) to assess the migration of the cells to the wounded region. Then, the images of the wound were analyzed using ImageJ software (NIH, United States).

### Cell migration experiments (Transwell)

Transwell chamber with matrix gel (Corning, United States) was prepared for the cell invasion test. After the starved MDA-MB-231 cells were resuspended in the sterile, serum-free DMEM medium, and inoculated to the upper chamber (at the cell density of 1 × 10^5^/well) and lower chamber using a complete medium with 5% FBS, and further incubated for 48 h with varying concentrations of AIL (0, 0.625, and 1.25 μmol/L), respectively. Thereafter, the cells were fixed with paraformaldehyde (4%), stained using gentian violet dye, dried, and observed under the optical microscope (Nikon, Japan). Micrographs were acquired from three randomly selected visual fields and counted by ImageJ software.

### Clone formation experiment

MDA-MB-231 cells were cultivated into the 6-well plate (at the cell densities of 1 × 10^4^/well), and varying concentrations of AIL (0, 0.625, and 1.25 μmol/L), respectively. The plates were incubated at 37°C for 14 days, and the solution was changed every 2 days. Thereafter, the cells were fixed using paraformaldehyde (4%), stained using gentian violet dye, dried, and micrographed under the optical microscope (Nikon, Japan) to estimate the number of colonies.

### Collection of MDA-MB-231 medium

In this study, experiments were also conducted to detect the influence of cytokines secreted by tumor cells on osteoclast differentiation. DMEM complete medium was used for culturing the MDA-MB-231 cells and the cells were inoculated in a 10 cm culture dish with 2 × 10^6^. After reaching a 90% convergence degree, the cells were incubated with varying AIL concentrations (0, 0.625, and 1.25 μmol/L) for 48 h. The medium was exchanged with the serum-free DMEM and cells were allowed to cultivate. After 48 h, the cell-free supernatant was separated and filtered using a 0.22 µm needle filter. The conditioned medium was divided into equal parts to avoid constant freezing and thawing, which could lead to denaturation. The different vials were stored at −80°C to be used within 3 months. For using the medium, it would be supplemented with fresh α- MEM medium (1:1 ratio), mixed, and used as a conditioned medium.

### Osteoclast differentiation *in vitro*

The logarithmically growing BMMs cells were digested, counted, and inoculated into the 96-well culture plates (8 × 10^3^/well), and osteoclasts were induced using three methods. RANKL (50 ng/ml) and M-CSF (20 ng/ml), RANKL (25 ng/ml) and M-CSF (20 ng/ml), or RANKL (25 ng/ml), M-CSF (20 ng/ml), and MDA-MB-231CM (using the same collection method used above). Varying concentrations of AIL (0, 0.625, and 1.25 μmol/L) were added to induce osteoclasts for differentiating the cells for 5 days. Based on the instructions printed on the Tartrate-resistant Acid Phosphatase (TRAP) staining kit (Sigma Aldrich, United States), BMMs cells in the control and drug treatment groups were stained using TRAP.

### Detection of F-actin ring of mature osteoclasts *in vitro*

BMMs cells (8 × 10^3^/well) was inoculated on 96 well plate, using RANKL (50 or 25 ng/ml), M-CSF (20 ng/ml), and MDA-MB-231CM (using the same collection method used above). Varying concentrations of AIL (0, 0.625, and 1.25 μmol/L) were added to induce osteoclast differentiation for 7 days. The cells were fixed with paraformaldehyde (4% v/v), at Room Temperature (RT) for 10 min. Then, cells were incubated with Triton X-100 (5% v/v) for 5 min, rinsed using PBS, and incubated with iFluor562-labeled phalloidin for in dark, for 30 min, at 4°C. These cells were rinsed using PBS and incubated with DAPI stains for 5 min, at RT, in dark. They were washed again with PBS and assessed under the fluorescent microscope (Nikon, Tokyo, Japan). Micrographs were obtained and the images were studied using the ImageJ software to determine the number of nuclei and F-actin rings in the osteoclasts.

### Bone absorption tests

Bone resorption tests were employed to assess osteoclast functions. The BMM cells (1 × 10^5^/well) were inoculated into the 24-well bone plate produced by Corning Company, in the presence of RANKL (50 or 25 ng/ml), M-CSF (20 ng/ml), and MDA-MB-231CM (using the same collection method used above). Varying concentrations of AIL (0, 0.625, and 1.25 μmol/L) were added for inducing osteoclast differentiation for 5 days. Thereafter, bone slices were obtained and stained using toluidine blue. Finally, the resorption area (%) in these bone slices was analyzed using the ImageJ software (NIH, United States).

### Enzyme-linked immunosorbent assay (ELISA)

Cytokines secreted by BC cells stimulate osteoclast differentiation in BMMs cells. The capacity of the BC cells to secrete certain cytokines after AIL treatment can be detected by ELISA. The MDA-MB-231 cells were digested and inoculated into the 6-well culture plate (1 × 10^6^/wells) and incubated at 37°C, overnight, in the 5% CO_2_ incubator until the cells adhered to the wall. Varying concentrations of AIL (0, 0.625, and 1.25 μmol/L) were added after 48 h and the cells were cultivated using the serum-free DMEM medium. The supernatant was collected after 48 h and the human RANKL ELISA kit was used to determine the concentration of IL-1β and RANKL. All steps were followed based on the instructions printed on the kit.

### RNA extraction and real-time fluorescence quantitative PCR

TRIzol reagent (AB Invitrogen, United States) was employed for extracting the total RNA content in the MDA-MB-231 cells treated using varying concentrations of AIL (0, 0.625, and 1.25 μmol/L). This same technique was implemented for extracting total RNA content from the BMM cells treated using RANKL (50 or 25 ng/ml) and MDA-MB-231CM (using the same collection method used above) after intervention using varying concentrations of AIL (0, 0.625, and 1.25 μmol/L). Total RNA (1 ug) was reverse transcribed into cDNA with the RT-PCR kit (Invitrogen, United States). The following conditions were used for RT-PCR in the study: 5 min reaction at 94°C, 30 cycles at 94°C, 40 s at 60°C, 40 s at 72°C, and final extension step at 72°C for 5 min. ViiA™ seven real-time fluorescence quantitative PCR apparatus (Applied Biosystems, United Kingdom) was used for this reaction. The Cycle threshold (Ct) value was determined and GAPDH was taken as an internal parameter 2^^−ΔΔCT^ technique was employed for data analysis. Table 1 lists the primers employed in this study.

**TABLE 1:** A List of primer sequences used for RT-PCR in the study.

Gene	Forward (5′–3′ sequence)	Reverse (5′–3′ sequence)
TGFβ	GGA​GAG​TGC​AGA​ACC​GGA​G	TCG​TTG​TGG​GTT​TCC​ACC​AT
CDH11	TGC​CTG​AGA​GGT​CCA​ATG​TG	TGG​GTA​GGG​CTG​TTC​TGA​TG
RUNX2	AGG​CAG​TTC​CCA​AGC​ATT​TCA	GGC​GGG​GTG​TAA​GTA​AAG​GT
CXCR4	TGT​TGT​CTG​AAC​CCC​ATC​CTC	GTC​CAC​CTC​GCT​TTC​CTT​TG
FOXP3	CTA​CCT​GGA​GAC​CTA​CGG​CG	TAT​AAA​GTG​CAG​GCC​CTG​GTG
GAPDH (human)	ATGGCACCGTCAAGGCTG	AGC​ATC​GCC​CCA​CTT​GAT​TT
CTSK	TAG​CAC​CCT​TAG​TCT​TCC​GC	CTT​GAA​CAC​CCA​CAT​CCT​GC
ACP5	TGG​GTG​ACC​TGG​GAT​GGA​TT	AGC​CAC​AAA​TCT​CAG​GGT​GC
NFATc1	CCA​GCT​TTC​CAG​TCC​CTT​CC	ACT​GTA​GTG​TTC​TTC​CTC​GGC
MMP9	GGC​ACC​ACC​ACA​ACA​TCA​CC	GGC​AAA​GGC​GTC​GTC​AAT​CA
DCSTAMP	GCT​GTA​TCG​GCT​CAT​CTC​CT	AAG​GCA​GAA​TCA​TGG​ACG​AC
GAPDH (mouse)	AGG​AGA​GTG​TTT​CCT​CGT​CC	TGA​GGT​CAA​TGA​AGG​GGT​CG

### Western blotting experiments

The effect of AIL on osteoclast formation associated proteins (TGF-β, PTHrP, MMP9, CXCR4) of the cultured MDA-MB-231 cells was determined by Western blotting experiments. MDA-MB-231 cells (5 × 10^5^/well) were inoculated in the 6-well plate, treated using varying concentrations of AIL (0, 0.625, and 1.25 μmol/L) for 48 h, and incubated in the presence of RIPA lysis buffer solution (Teye, Bioteke Cor., China) at 4°C to lyse the cells and extract the total protein. Similarly, the effect of AIL on FOXP3 protein in the MDA-MB-231 cells was evaluated by the same method. Western blotting experiments were used for determining the effect of AIL on the PI3K/AKT, NF-κB, and MAPK signaling pathways in the MDA-MB-231CM-induced BMMs cells. BMMs cells (4 × 10^4^/well) were inoculated on a 6-well plate, eight wells in total were necessary and split into two groups, where Group 1 was treated using MDA-MB-231CM, while Group 2 was treated with AIL (1.25 μmol/L) AIL intervention of MDA-MB-231CM. The phosphorylation levels of JNK, AKT, IκBα, P65, ERK, P38, and PI3K were observed by protein blotting at various time points (0, 15, 30, and 60 min). Western blotting experiments were implemented to assess the impact of AIL on the osteoclast-specific proteins (MMP9, NFATc1, CTSK, TRAP, DCSTAMP) induced by RANKL (25 ng/ml), and MDA-MB-231CM. BMM cells were inoculated in a 6-well plate (6 × 10^5^/well), RANKL (25 ng/ml) and MDA-MB-231CM with different AIL concentrations (0, 0.625, and 1.25 μmol/L) after intervention (using the same collection technique used above) was used for inducing the differentiation of BMM cells, and the protein samples were collected for 5 days and assessed using Western blotting.

### Animal experiments

The Xuanzhu Biological Science Company (Hangzhou, China) provided the 4 week-old BALB/c female, nude mice, which were then split randomly into three groups (normal saline, 10, and 15 mg/kg groups, n = 5). The right tibial medullary cavity of the mice was directly injected with MDA-MB-231-Luc cells (2 × 10^5^ cells suspended in 20 μl PBS). The animals underwent intraperitoneal drug injections every other day beginning on Day 2 after the injection with MDA-MB-231-Luc cells, and they were euthanized after 6 weeks of the intervention treatment. Throughout the experiment, the body weights of all animals were estimated after every 4 days. Right tibia samples were extracted for micro computed tomography imaging (μCT), histological analysis, and tumor size measurement.

### IVIS imaging

Each week, MDA-MB-231-Luc cell development in the tibia of each mouse was monitored by VIS imaging (Xenogen, MA, United States). Isoflurane gas (2% isoflurane in oxygen, 1 L/min) was used to anesthetize all animals before imaging. Following intraperitoneal injection of 100 μl D-luciferin solution (15 mg/ml), each animal was scanned after 15 min, and bioluminescence images were then collected. A circular ROI was drawn around every biological light source with the help of the Living Image software (Xenogen Corp.), which was then used to estimate the signal. These results were presented as the total photon flux in the ROI, and were measured and expressed as photons per second (photons × s) ^−1^ × cm^−2^ × sr^−1^.

### Micro-computed tomography scanning

The trabecular bone samples from the proximal tibia were scanned under conditions of 500 μA current and 50 kV tube voltage. Then, 2-Dimensional (2D) and 3D structures of proximal tibia samples were reconstructed using the Mimics 18.0 software (Materialise, Belgium). The following histological parameters of the proximal tibia were examined using a plug-in program: Trabecular number (Tb. N), Bone Mineral Density (BMD), Trabecular separation (Tb. Sp), Bone Volume/Tissue Volume (BV/TV), and Trabecular thickness (Tb. Th).

### Bone histological analysis

The left lower limbs of the mouse models were surgically removed, fixed using formaldehyde (4%) at RT, and decalcified using the aqueous Tetrasodium Ethylenediaminetetraacetic Acid (T-EDTA) solution (10% v/v) for 2 weeks. The tissues were embedded into liquid paraffin molds and sectioned into 4 mm thick slices using a microtome. Hematoxylin-eosin (H&E) staining procedure was implemented to evaluate the trabecular bone, while the RANKL, TRAP, and IL-1β concentrations were analyzed using Immunofluorescence (IF) staining. FOXP3 concentrations were detected using immunohistochemistry. Standard bone section measurements were assessed using the microscope, and data were assessed using the ImageJ software (NIH).

### Statistical analysis

Every experiment was conducted in triplicates. Statistical analysis and charts were assessed with the help of the GraphPad Prism ver. 9.0 software (GraphPad software, United States). The results have been described as mean ± Standard Deviation (SD) for the three cell experiments and five animal-based experiments. The *t*-test was employed for pairwise comparisons between all the groups, and univariate analysis of variance was applied for comparing the data derived from different groups. Values with *p* < 0.05 were statistically significant (*p < 0.05, **p < 0.01, ***p < 0.001, ****p < 0.0001).

## Results

### AIL inhibits the *in vitro* migration, infiltration, and colony formation of the MDA-MB-231 cells

**FIGURE 1:**
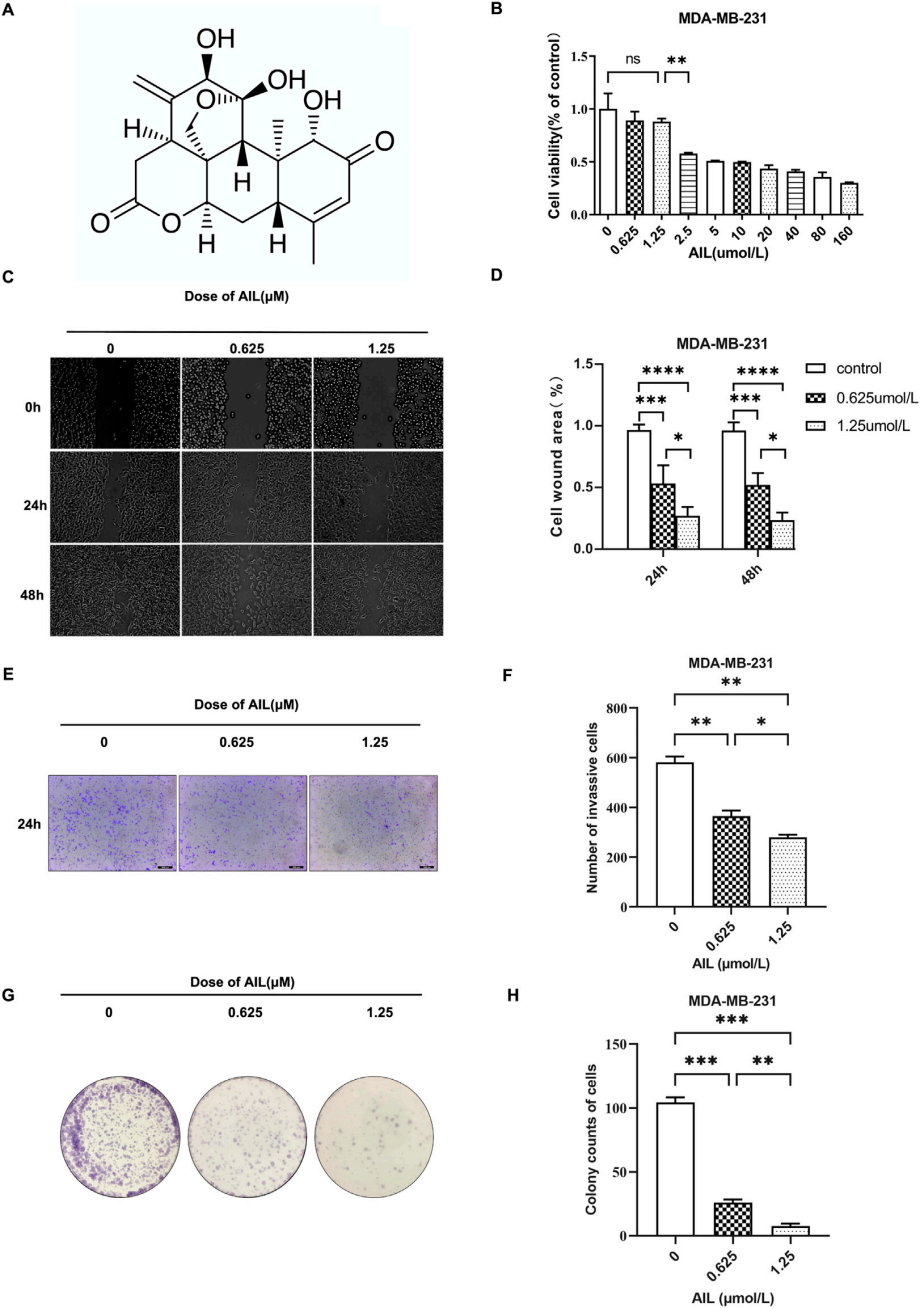
Non-toxic concentrations of AIL inhibit the migration, infiltration, and cloning capacity of the MDA-MB-231 cells, during the *in vitro* experiments. **(A)** Chemical structure of AIL. **(B)** Cytotoxicity of the MDA-MB-231 cells after being treated with varying AIL concentrations for 48 h. **(C,D)** Images presenting the migratory potential of MDA-MB-231 cells following 24 –48 h of treatment using differing concentrations of AIL (100 × magnification). The ImageJ software was used to calculate the wound healing percentage (%). **(E,F)** Images depicting the invasiveness of MDA-MB-231 cells 24 h after treatment using varying AIL concentrations (100 × magnification). Cells that invaded the body were counted. **(G,H)** A whole-hole image depicting the capacity of MDA-MB-231 cells to form colonies after being exposed to varying concentrations of AIL. Cells that invaded the body were counted. The mean ± SD of the three replicates were used to express all the data. *p < 0.05, **p < 0.01, ***p < 0.001, ****p < 0.0001 in comparison to the control animals.

Figure 1A depicts the chemical structure of AIL. MDA-MB-231 cells were used to determine the cytotoxicity of AIL. Results from the CCK8 test revealed that the 48 h OS rate of MDA-MB-231 cells was unaffected by AIL (0.625 and 1.25 μmol/L concentrations) (Figure 1B). Migration and infiltration ability of the malignant cells was the primary factor that led to bone metastasis in BC. Hence, in this study, the effect of AIL in inhibiting the migrating and infiltration capacity of the MDA-MB-231 cells was assessed. The wound healing experiment demonstrated that the MDA-MB-231 cells showed a significant decrease in their migratory ability at 24 and 48 h in a concentration-dependent manner when the AIL concentrations were ≥0.625 μmol/L **(** Figures 1C, D). The transwell experimental results indicated that AIL significantly suppressed the infiltration of MDA-MB-231 cells at concentrations of 0.625 μM or 1.25 μM. Thus, it could be concluded that this inhibition was positively linked with the varying concentrations of AIL (Figures 1E, F). Cloning, in addition to the migration and invasion steps, was also involved in the transmission of BC cells. As a result, colony formation experiments were undertaken to determine the influence of AIL on the ability of MDA-MB-231 cells for *in vitro* cloning (Figure 1G). The results demonstrated that treatment with AIL at 0.625 μM or 1.25 μM substantially lowered the capacity of MDA-MB-231 cells to form colonies in a concentration-dependent manner (Figure 1H).

### AIL downregulated the expression of the osteoclast formation-associated genes and protein markers in MDA-MB-231 cells

**FIGURE 2:**
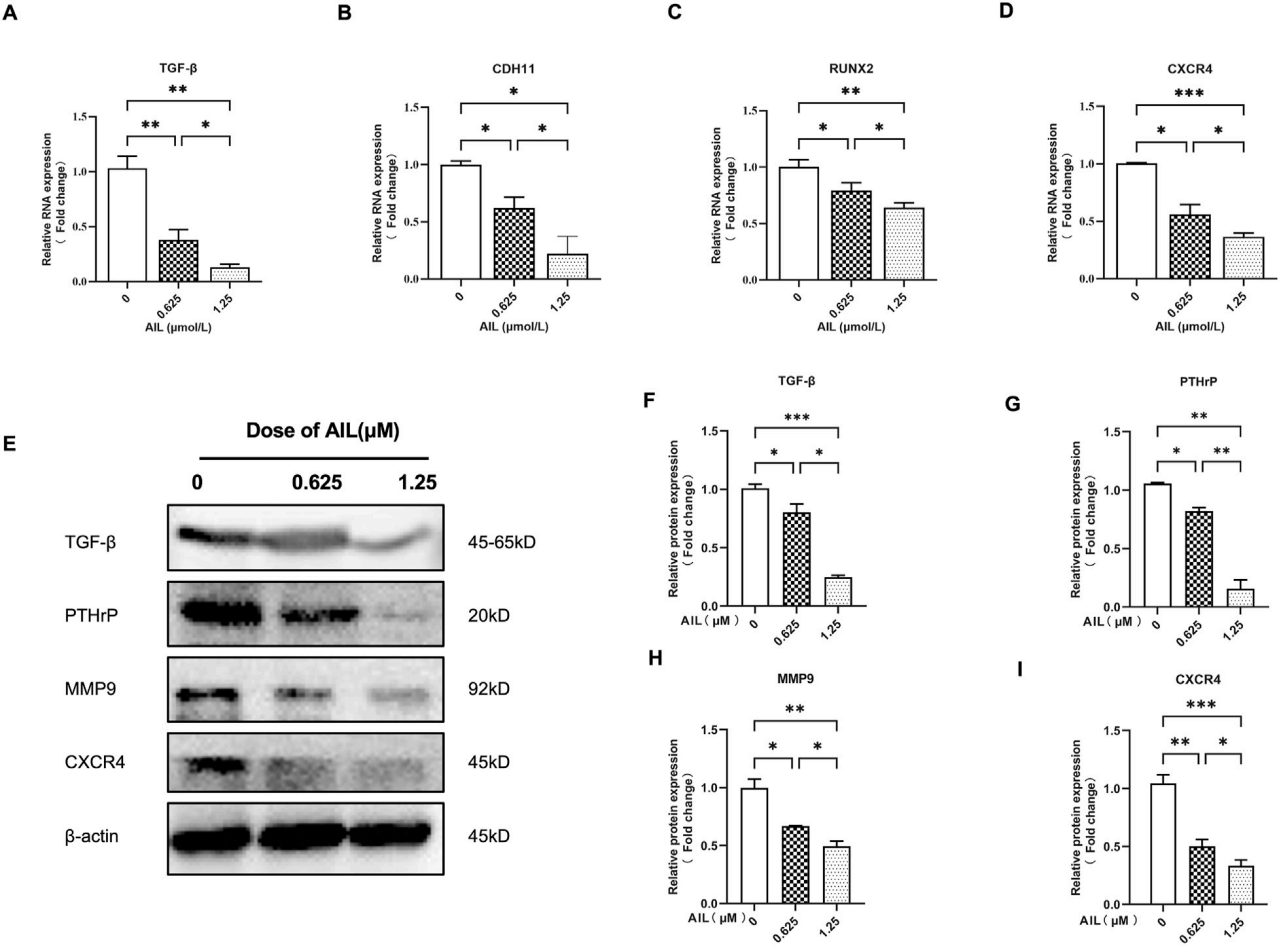
In MDA-MB-231 cells, AIL downregulates the expression levels of the genes and protein markers linked to osteoclast development. **(A–D)** The MDA-MB-231 cells were treated using varying AIL concentrations, RNA was collected, and qPCR analysis indicated that the expression levels of TGF-β, CDH11, RUNX2, CXCR4 genes involved in osteoclast formation was down-regulated. The 2^^ΔΔCT^ method was utilized to evaluate the data, and Table 1 contains a list of the individual primers that were employed. **(E)** After subjecting MDA-MB-231 cells to differing AIL concentrations, the total protein content was extracted, and Western blot analysis was employed to identify the expression levels of TGF-β, PTHrP, MMP9, CXCR4, the protein markers related to osteoclast formation. **(F–I)** Western blotting experiments were used to determine the osteoclast-related protein expression following treatment with various doses of AIL. The mean ± SD of the three replicates were used to express all the data. *p < 0.05, **p < 0.01, ***p < 0.001, in comparison to the control animals.

Some overexpressed genes and proteins in BC cells are associated with the invasiveness of bone metastasis and the increase of osteoclast activity, including TGF-β, CDH11, RUNX2, CXCR4, PTHrP, and MMP9 (Sethakorn et al., 2022). Therefore, it was determined how AIL affected the protein and gene expression levels in the MDA-MB-231 cells, which could help to explain the bone metastasis capacity of the BC cells. Table 1 lists the nucleotide sequences corresponding to a gene. The findings revealed that the AIL concentrations of 0.625 μM or 1.25 μM, significantly suppressed the expression of the TGF-β, CDH11, RUNX2, and CXCR4 genes (Figures 2A–D). At the same time, the TGF-β, PTHrP, MMP9, and CXCR4 protein levels were also determined. The results revealed that AIL inhibited the protein expression levels in a concentration-dependent manner (Figures 2E, F–I).

### AIL inhibited osteoclastogenesis induced by both RANKL and MDA-MB-231 CM

**FIGURE 3:**
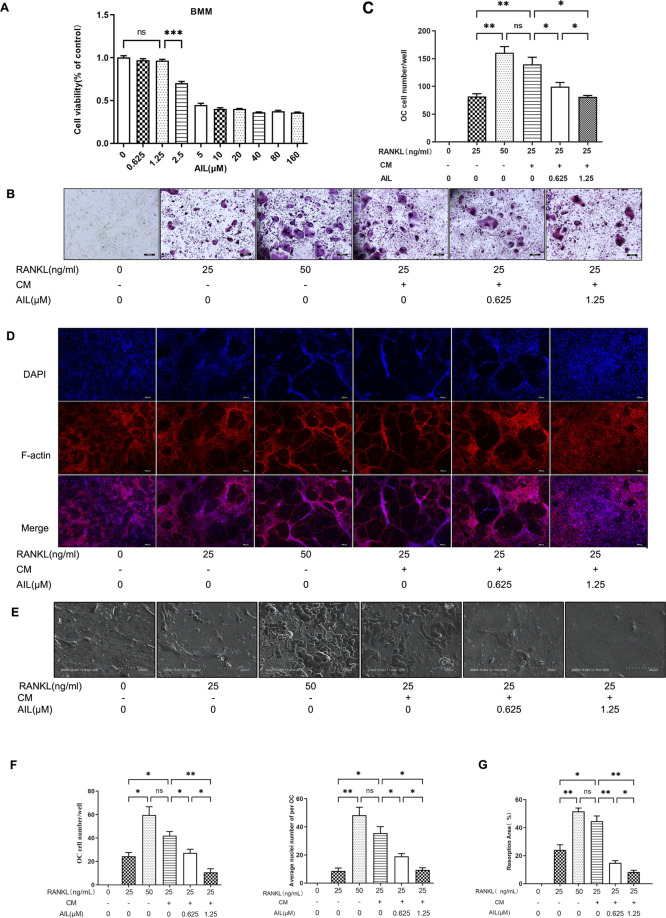
The supernatant of MDA-MB-231 cells can increase the osteoclast differentiation induced by RANKL, and non-toxic concentration AIL can block the osteoclast differentiation and its function simultaneously induced by RANKL and MDA-MB-231CM *in vitro*. **(A)** Cytotoxicity of BMM cells after 48 h of exposure to various AIL concentrations. **(B,C)** Different RANKL concentrations (25 or 50 ng/ml) were used to generate TRAP staining images (50 × magnification) of the BMM cells and quantitative results of TRAP-positive multinuclear cells were determined. The MDA-MB-231CM were treated using varying AIL concentrations. **(D,F)** An image (200 × magnification) of actin ring formation, induced by varying concentrations of RANKL (25 ng/ml or 50 ng/ml) and MDA-MB-231CM treated with differing AIL concentrations, was obtained using immunofluorescence in combination with intranuclear DAPI staining. Both the total no. Of osteoclasts and average no. Of nuclei in each osteoclast were determined. **(E,G)** AIL treatment at various dosages decreased bone absorption in the BMM cells that were treated using MDA-MB-231CM and varying RANKL concentrations (25 or 50 ng/ml) (200 × magnification). The ImageJ software was employed to calculate the area of bone absorption pits in the above image. The mean ± SD of the three replicates were used to express all the data. *p < 0.05, **p < 0.01, ***p < 0.001, in comparison to control animals.

The cytotoxicity of AIL (0.625, 1.25, 2.5, 5.0, 10, 20, 40, 80, and 160 μM) to BMM cells was determined by CCK-8 method. The cell optical density was tested at 450 nm after 48 h of treatment with AIL, and the data showed that when the concentration was less ≤1.25 µM, AIL did not show any obvious cytotoxic effect on BMM cells (Figure 3A). Some cytokines secreted by BC cells are seen to promote osteoclast precursor cells to differentiate into osteoclasts by activating specific signal pathways, thus enhancing pathological osteolysis (Méndez-García et al., 2019). In an earlier study, the results showed that the treatment strategy involving 50 ng/ml RANKL + 20 ng/ml M-CSF, induced the BMM cells to form osteoclasts (Zhong et al., 2019). To determine whether AIL blocks or slows down the differentiation of osteoclasts induced by BC cells, the conditioned medium of the MDA-MB-231 cells was collected and the RANKL concentration was decreased to 25 ng/ml, and osteoclasts were induced using 20 ng/ml M-CSF and MDA-MB-231 CM. After treatment with AIL, TRAP staining process was utilized to evaluate the impact of AIL on the differentiation of osteoclasts induced by MDA-MB-231 cell-conditioned medium. The findings of these experiments showed that no. Of TRAP-positive cells, induced by 20 ng/ml M-CSF and 25 ng/ml RANKL, were seen to be lesser compared to those stimulated by 20 ng/ml M-CSF and 50 ng/ml RANKL, and this difference was seen to be statistically significant. However, in the 20 ng/ml M-CSF and 25 ng/ml RANKL groups, the no. Of TRAP-positive cells induced by adding MDA-MB-231 CM increased significantly, and there was no statistical difference with the 20 ng/ml M-CSF and 50 ng/ml RANKL groups, which indicated that adding MDA-MB-231 CM could promote the BMM cell differentiation into osteoclasts. Different concentrations (0, 0.625, and 1.25 µM) of AIL can significantly reduce the osteoclast differentiation stimulated by 20 ng/ml M-CSF, 25 ng/ml RANKL, and MDA-MB-231 CM, and the no. Of TRAP-positive cells could be significantly reduced in a concentration-dependent manner (Figures 3B, C).

### AIL inhibits the formation of F-actin ring in osteoclasts that were induced by RANKL and MDA-MB-231 CM

The most visible characteristic of osteoclasts to absorb bone is the F-actin ring, which is perceived to be a form of cytoskeleton structure. The influence of AIL on the osteoclast function was determined by assessing if the RANKL and MDA-MB-231 CM-induced AIL influenced the development of osteoclast fibroactin (F-actin) ring in the osteoclasts. The actin ring structure was identified using DAPI and phalloidin staining. The osteoclasts activated by the 20 ng/ml M-CSF and 25 ng/ml RANKL group produced fewer actin rings and nuclei than those induced by 20 ng/ml M-CSF and 50 ng/ml RANKL, and this difference was seen to be statistically significant. However, adding the MDA-MB-231 CM to the 20 ng/ml M-CSF and 25 ng/ml RANKL groups significantly increased the quantity of actin rings and nuclei in osteoclasts, whereas no statistically-significant difference was noted in the 20 ng/ml M-CSF and 50 ng/ml RANKL groups. The outcomes also demonstrated that the addition of MDA-MB-231 CM may encourage osteoclast differentiation in BMM cells. The quantity of actin rings and nuclei in osteoclasts generated by BMM cells and induced by RANKL and MDA-MB-231CM significantly decreased after treatment with differing concentrations of AIL (0, 0.625, and 1.25 μM). The findings demonstrate that AIL inhibits the development of actin rings in mature osteoclasts (Figures 3D–F).

### AIL inhibits the bone resorption function of the RANKL and MDA-MB-231 CM-induced osteoclasts

To confirm whether AIL inhibits the bone absorption function of the osteoclasts that were co-induced by MDA-MB-231 CM, bone slices were deposited in the 48 well culture plates and mature osteoclasts were inoculated in these wells. Then, different concentrations of AIL (0, 0.625, and 1.25 μM) were added to the treatment groups, along with M-CSF (20 n/ml), RANKL (25 ng/ml), and MDA-MB-231 CM. The control groups did not contain AIL. To identify resorption pits, toluidine blue was used to stain bone slices after 48 h. The findings demonstrated that the area of absorption pits noted on the osteoclast bone slice surfaces stimulated by 20 ng/ml M-CSF and 25 ng/ml RANKL were less than those induced by 20 ng/ml M-CSF and 50 ng/ml RANKL, and this difference was seen to be statistically significant. No statistical difference was noted between the 20 ng/ml M-CSF and 50 ng/ml RANKL groups. However, the area of absorption pits on the osteoclast bone slice surfaces in the animals from the 25 ng/ml RANKL and 20 ng/ml M-CSF groups (that were induced after the MDA-MB-231 CM addition) increased significantly. The findings demonstrated that the MDA-MB-231 CM addition improved the bone absorption ability of the osteoclasts. However, following the treatment with varying concentrations of AIL (0, 0.625, and 1.25 μM), the depression development on the osteoclast bone slices produced by BMM cells and stimulated by RANKL and MDA MB-231CM was inhibited significantly (Figure 3E). To assess the osteoclasts’ ability to absorb bone, the proportion of absorption pits on the bone slice surfaces was compared between the treatment and control groups. Results have been expressed in Figure 3G, where it was noted that AIL reduced the ability of mature osteoclasts to produce pits on their hydroxyapatite-coated plates.

### AIL inhibits osteoclast-related gene and protein expression induced by RANKL and MDA-MB-231CM

**FIGURE 4:**
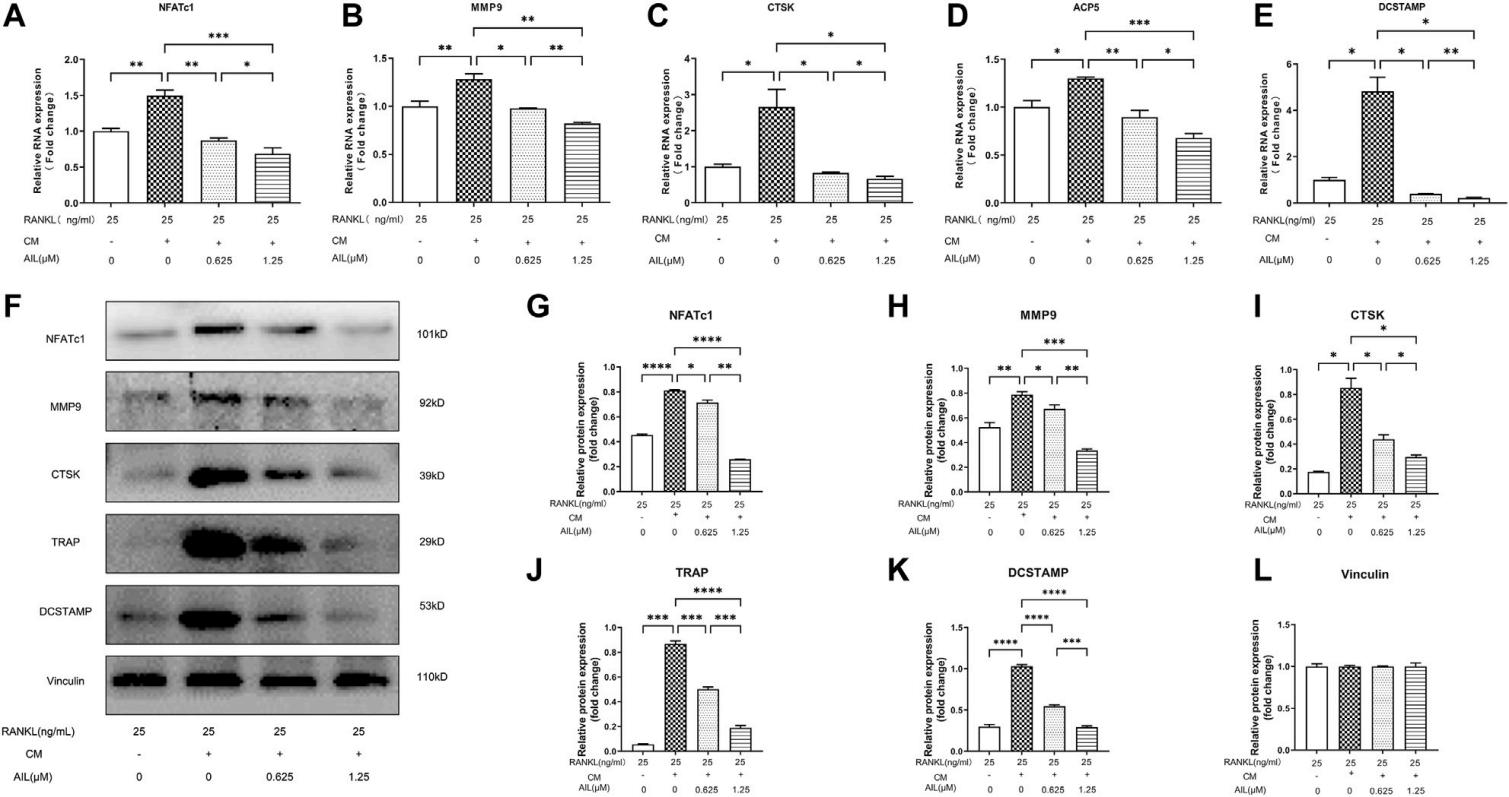
In BMM cells, AIL suppresses the expression levels of the osteoclast-generating genes and protein markers that were simultaneously stimulated by RANKL and MDA-MB-231CM. **(A–E)** RNA was extracted from BMM cells that had been exposed to various concentrations of AIL, and qPCR was used to determine if the osteoclast-producing genes such as NFATc1, CTSK, MMP9, ACP5, and DCSTAMP had been downregulated. The 2^^ΔΔCT^ method was utilized to evaluate the data, and Table 1 presents the list of individual primers used in the study. **(F)** The total protein content was extracted after MDA-MB-231CM treated using various doses of AIL was used to induce BMM cells. Western blot analysis was employed for evaluating the expression of osteoclast-producing protein markers like NFATc1, MMP9, CTSK, TRAP, and DCSTAMP. **(G–L)** Western blotting experiments were conducted to determine the expression levels of the osteoclast-related proteins following treatment with various doses of AIL. The mean ± SD of the three replicates were used to express all the data. *p < 0.05, **p < 0.01, ***p < 0.001, ****p < 0.0001, in comparison to the control animals.

The results implied that the expression levels of the associated marker genes like CTSK, ACP5, NFATc1, and MMP9 (Table 1 presents their nucleotide sequences) were associated with the differentiation and functionality of osteoclasts. The impact of AIL on the gene expression of osteoclasts induced by M-CSF (20 ng/ml), RANKL (25 ng/ml), and MDA-MB-231CM was assessed using the real-time fluorescent quantitative PCR technique. BMM cells that were induced with RANKL (25 ng/ml), M-CSF (20 ng/ml), and MDA-MB-231 CM were treated using differing AIL concentrations (0, 0.625 and 1.25 μM) for 5 days. The findings of the experiment revealed that AIL significantly reduced the upregulation of CTSK, ACP5, NFATc1, DCSTAMP, and MMP9 gene expression induced by MDA-MB-231 CM and RANKL (Figures 4A–E). Thus, it was concluded that AIL significantly inhibited the upregulation of osteoclast-related gene expression induced by RANKL and MDA-MB-231 CM.

Numerous marker proteins, including NFATc1, MMP9, CTSK, TRAP, DCSTAMP, etc., are expressed in association with osteoclast differentiation and function. Therefore, Western blotting experiments were used to assess the effect of AIL on CTSK and other related proteins stimulated by M-CSF (20 ng/ml), RANKL (25 ng/ml), and MDA-MB-231 CM. The findings demonstrated that the concentration-dependent addition of AIL reduced protein expression levels (Figures 4F–L). According to PCR analysis and Western blot data, AIL suppressed the expression levels of osteoclast-specific genes that were stimulated by MDA-MB-231 CM and RANKL, decreased the level of osteoclast-specific protein, and prevented the osteoclast differentiation of BMM cells.

### AIL inhibited the activation of PI3K/AKT, NF-κB, and MAPK signaling pathways that were induced by MDA-MB-231 CM

**FIGURE 5:**
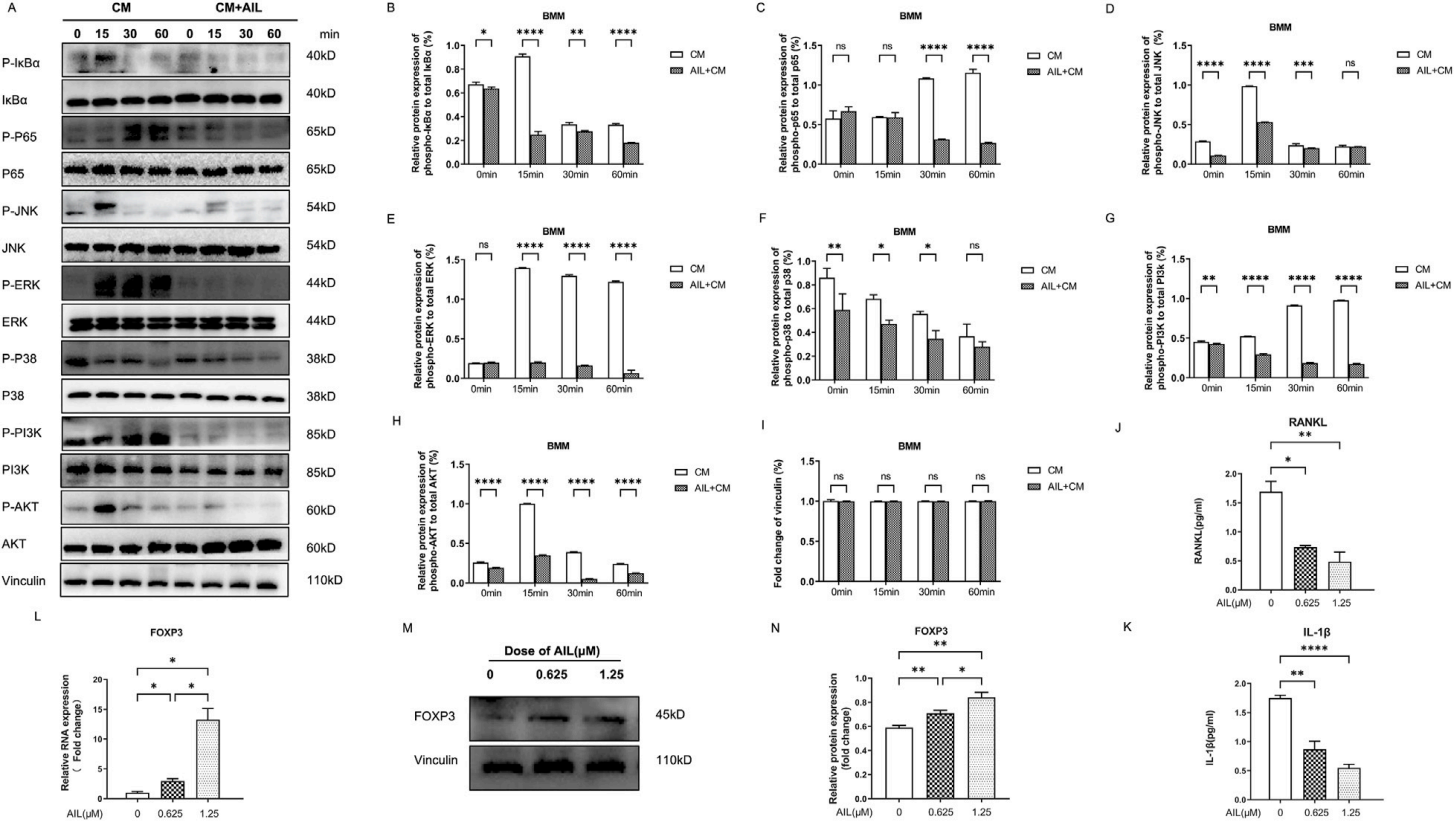
AIL inhibits the stimulation of the MAPK, NF-κB, and PI3K/AKT signaling pathways, induced by MDA-MB-231CM, by suppressing the IL-1β and RANKL in MDA-MB-231CM. **(A)** BMM cells were induced to differentiate with AIL (1.25 μM) treated MDA-MB-231CM, and lysed cells to collect proteins at 0, 15, 30, and 60 min, respectively. The phosphorylation of the major proteins involved in the NF-κB pathway, including IκBα and P65, was detected using the Western blotting technique. The phosphorylation of major proteins like AKT and PI3K in the PI3K-AKT signaling pathway, was detected using Western blotting experiments. The phosphorylation level of the major proteins involved in the MAPK pathway, including JNK, ERK, and P38, were detected using Western blots. **(B–I)** Quantitative phosphorylation and total IκBα, p65, PI3K, AKT, JNK, ERK, and P38 protein expression levels. **(J,K)** The IL-1β and RANKL expression levels in the cell-free supernatant of MDA-MB-231 cells treated using differing AIL concentrations were examined. **(L)**. When the MDA-MB-231 cells were treated using differing concentrations of AIL, RNA was extracted, and the FOXP3 gene expression was upregulated by qPCR analysis. The 2^^ΔΔCT^ technique was utilized to evaluate the data, and Table 1 provides a list of individual primers used in the study. **(M,N)** To ascertain the impact of various AIL concentrations on the FOXP3 protein expression levels in the MDA-MB-231 cells and its quantification, the Western blotting technique was used. The mean ± SD of the three replicates were used to express all the data. *p < 0.05, **p < 0.01, ***p < 0.0001, ****p < 0.0001, ns – no significance, in comparison to the control.

Osteoclast development requires the secretion of a few proteins by BC cells as well as the activation of the RANKL-induced MAPK, NF-κB, and PI3K-AKT signaling pathways (Song et al., 2022). To detect whether BMM cells induced by AIL could affect the MDA-MB-231 CM and inhibit the osteoclast generation that was mediated by NF-κB signaling pathway, the P65 and IκBα phosphorylation levels were assessed with the Western blot analysis. As depicted in Figure 5A, the IκBα and P65 phosphorylation were activated within 15, 30, or 60 min, respectively, under the stimulation of MDA-MB-231 CM. However, after induction of 1.25 μM AIL-treated MDA-MB-231 CM, the phosphorylation levels of these proteins were partially inhibited (Figures 5B, C). Therefore, the results indicated that AIL could inhibit the NF-κB signaling pathway activation that was induced by MDA-MB-231 CM.

The modulation of osteoclast proliferation and functions may also be influenced by the MAPK signaling pathway in addition to the NF-κB signaling system. Western blotting experiments were used to detect the phosphorylated ERK, JNK, and P38 proteins to assess whether AIL suppressed the MAPK pathway-mediated osteoclastogenesis. According to the findings, AIL treatment suppressed MDA-MB-231CM almost immediately after induction (Figures 5A, D–F). It showed that AIL prevented the activation of the MAPK pathway during development of osteoclasts, which were stimulated by MDA-MB-231 CM.

As downstream targets, PI3K and AKT signaling pathways are seen to play a vital role in differentiation and functioning of osteoclasts. In this study, experiments were carried out to evaluate if incubating the MDA-MB-231CM-treated AIL and BMM cells could inhibit osteoclast formation via the PI3K and AKT signaling pathways, and the Western blotting results were used to assess the phosphorylation levels of PI3K and AKT. As described in Figure 5A, the phosphorylation level of PI3K-AKT pathway was significantly enhanced after MDA-MB-231 CM stimulation. However, this phosphorylation was significantly reduced when treated using AIL (Figures 5G, H). There was no change in the protein vinculin (Figure 5I). Therefore, the above findings revealed that AIL inhibited the upregulation of PI3K-AKT signaling pathway induced by MDA-MB-231 CM.

### AIL inhibits the RANKL and IL-1β cytokine levels in MDA-MB-231 CM

To study which cytokines in MDA-MB-231 CM promote the differentiation of osteoclasts, ELISA tests of RANKL and IL-1β in MDA-MB-231 CM. The findings revealed that the treatment with 0.625 μM or 1.25 μM concentrations of AIL significantly decreased the RANKL and IL-1β levels in MDA-MB-231 CM (Figures 5J, K), indicating that the inhibition of AIL on the formation of osteoclasts co induced by RANKL in MDA-MB-231 CM may be partly attributed to its inhibition on the paracellular secretion of cytokines in MDA-MB-231 CM.

### AIL is involved in the upregulation of the FOXP3 gene and protein expression levels in MDA-MB-231 cells

In human BC, near Smooth Muscle Actin (SMA) stromal cells, a majority of T-cell that secreted RANKL were seen to express the FOXP3 transcription factor (Tan et al., 2011). To further investigate whether AIL inhibited the expression of the RANKL and IL-1β cytokines in MDA-MB-231CM through FOXP3, the FOXP3 gene and protein expression levels in MDA-MB-231 cells after AIL treatment were assessed. The findings of these experiments revealed that AIL significantly upregulated the FOXP3 gene and protein expression levels in MDA-MB-231 cells, which suggests that AIL may inhibit the RANKL and IL-1β secretion by upregulating the FOXP3 expression in MDA-MB-231 cells (Figures 5L–N).

### Effect of AIL on bone damage in mice with bone metastasis of BC

**FIGURE 6:**
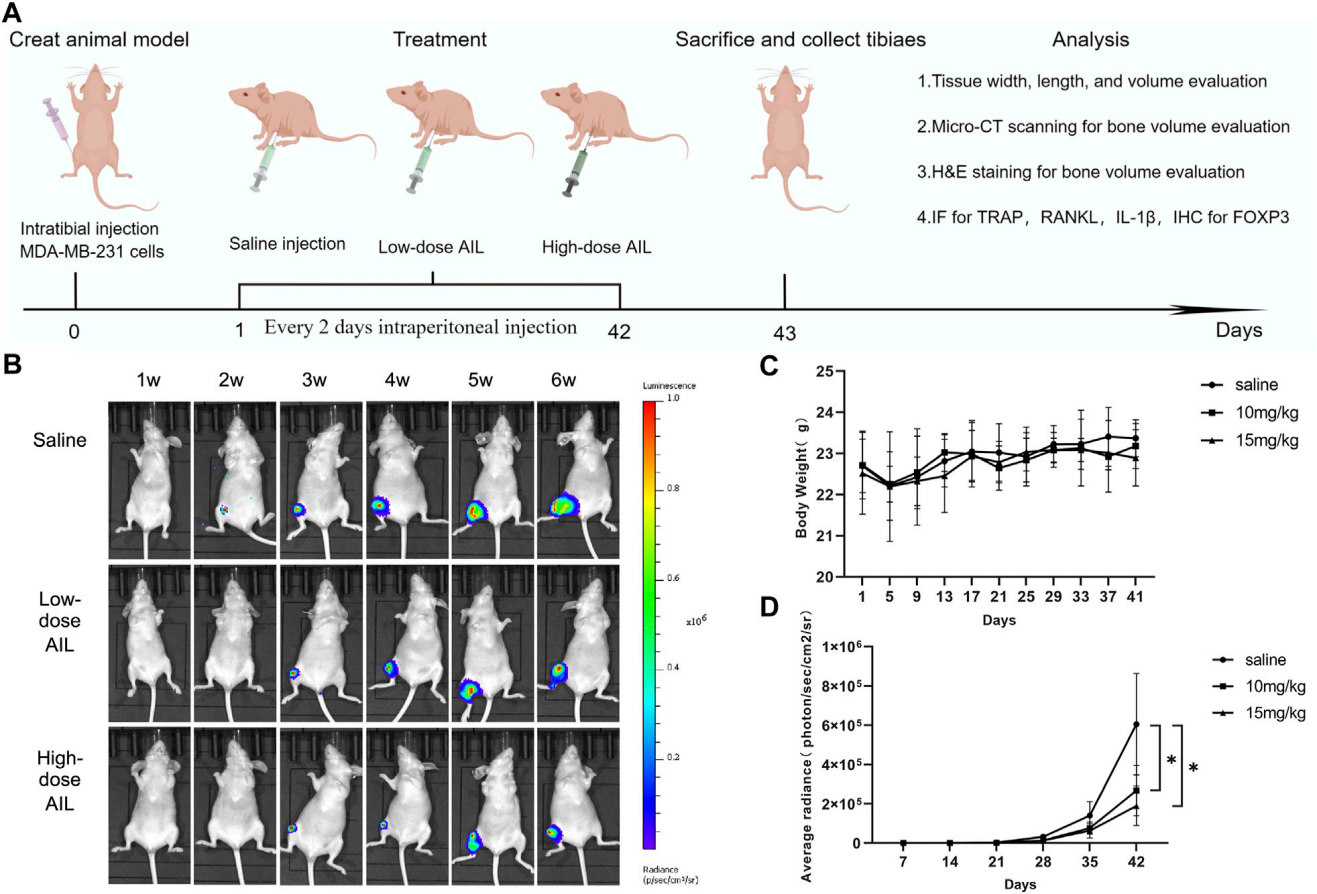
AIL prevents bone metastases and osteolysis brought on by BC and inhibits the evolution of the disease, *in vivo*. **(A)** The *in vivo* treatment timeline was created using the Figdraw software (ID: AIATW58585). A BC-induced bone metastasis model in mice was developed by injecting Luc cells into right tibial plateau of nude mice. **(B)** Non-invasive IVIS was used every week to identify the representative bioluminescence images for developing MDA-MB-231 Luc cells in the tibia of nude mice. **(C)** Every 4 days, the body weight variations in every group were assessed. **(D)** The graphs depicting the bioluminescence activity of tumor cells in the bone microenvironment with time. The mean ± SD were used to express all the data. *p < 0.05, **p < 0.01, in comparison to the control animals.

The findings revealed that AIL inhibited the osteoclasts that were stimulated by RANKL and MDA MB-231CM during the *in vitro* experiments. Hence, the *in vivo* activity of AIL in the mouse model with BC-induced bone metastasis needs to be investigated. *In situ* MDA-MB-231-Luc cell injection into the tibial medullary cavity was used to create a mouse bone metastases model (Figure 6A). Then, *in vivo* bioluminescence imaging was carried out once per week in the normal saline, the 10 mg/kg, and the 15 mg/kg groups, following the injection of MDA-MB-231-Luc cells. A representative bioluminescence image of bone metastasis progress is shown in Figure 6B. The total bioluminescence signal intensity of AIL treatment group was relatively weaker compared to that displayed by the normal saline group. The total bioluminescence signal intensity was used to evaluate the tumor load, as described in Figure 6D. In comparison with the normal saline group, the total tumor load was significantly reduced in the 10 and 15 mg/kg groups. During the complete experiment, no significant differences were noted in the body weights of nude mice in each group over time (*p* > 0.05) (Figure 6C). After 42 days, the bone metastasis tissue of tibial breast cancer was extracted (Supplementary Figure S1A) and the tumor formation was measured. The tumor growth in the normal saline group mice was rapid; however, the tumor growth was significantly reduced in the AIL treatment groups (Supplementary Figures S1B–D). These findings indicate that AIL effectively inhibited the *in vivo* progression of the BC bone metastasis cells.

### AIL regulates the RANKL and IL-1β secretion by the BC cells *via* the *in vivo* FOXP3 expression, for inhibiting the bone resorption and bone metastasis of osteoclasts

**FIGURE 7:**
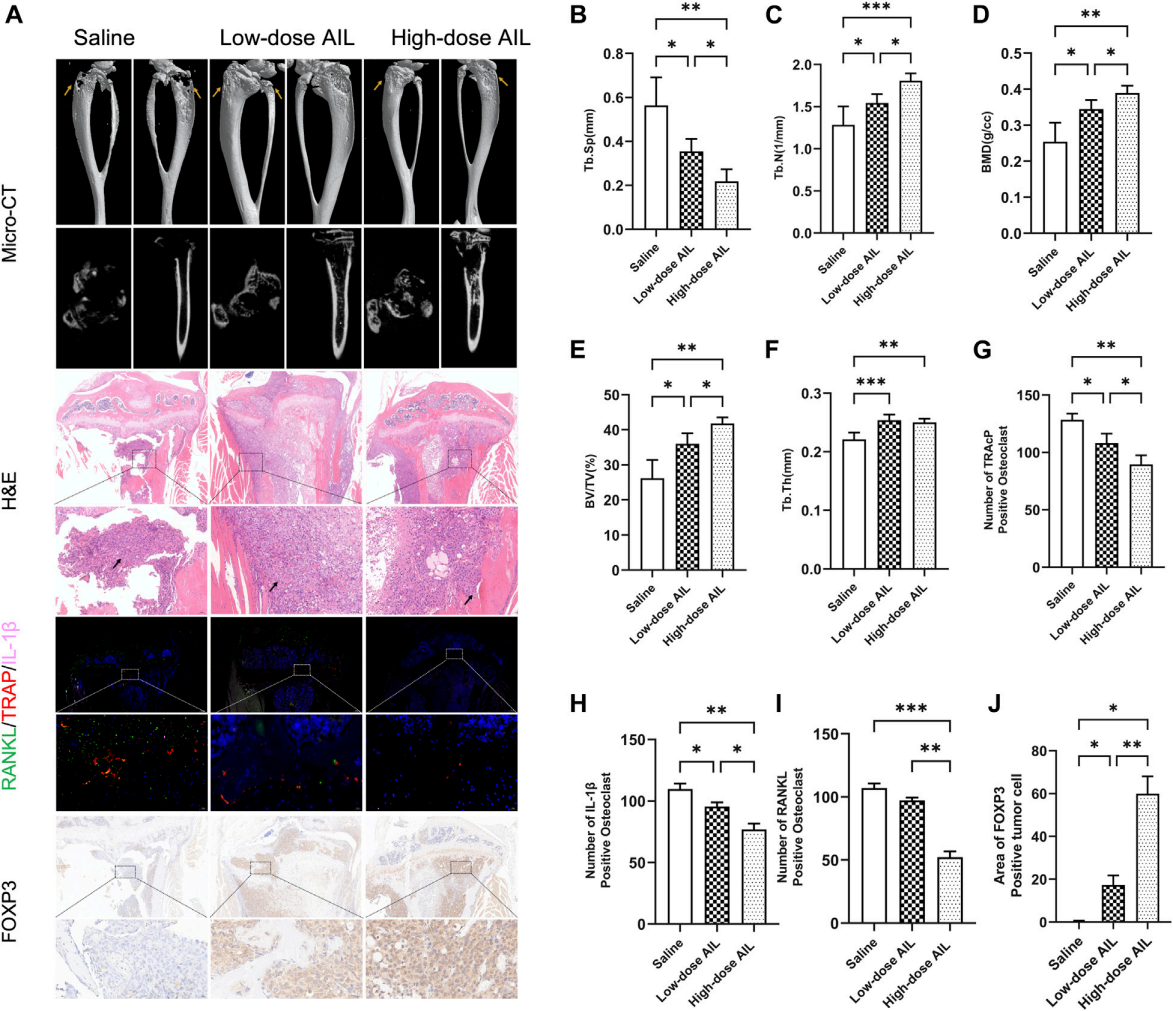
AIL controls RANKL and IL-1β released by BC cells through FOXP3 protein expression *in vivo* to prevent osteoclast development and bone metastases. **(A)** 3D reconstruction of microCT, H&E, biological staining of RANKL (green), TRAP (red), and IL-1β (pink) in the tibias tissue of mice in the tumor-bearing groups of BC, and representative images of the FOXP3 immunohistochemical staining results. The osteolytic lesion area is indicated by the yellow arrow, while the tumor area is indicated by the black arrow. **(B–F)** BV/TV, BMD, Tb. Th, Tb. Sp, and Tb. N. The quantitative analysis was carried out using the μCT Skycan CTAn software. The mean ± SD were used to express all the data. *p < 0.05, **p < 0.01, ***p < 0.001. **(G–I)** The TRAP, IL-1β and RANKL were quantitatively analyzed in proximal tibia of mice in the normal saline and AIL treatment groups **(J)** The FOXP3 positive cells were quantitatively analyzed in proximal tibia of three mice groups. The mean ± SD were used to express all the data. *p < 0.05, **p < 0.01, ***p < 0.001. Magnification of 5×/20× (left/right, H&E), 5×/40× (left/right, IF), 5×/40× (left/right, FOXP3 IHC).

Additionally, the results of the microCT technique showed that the AIL inhibited the BC-induced osteolysis. The findings demonstrated that mice in the normal saline group showed visible bone erosion on the inside and outside surfaces of tibia. AIL treatment (15 mg/kg) decreased the osteolysis on the outer and inner surface of tibia, while the 10 mg/kg treatment showed a marginal effect (Figure 7A). Bone Mineral Density (BMD), Bone volume fraction (BV/TV), Trabecular separation (Tb. Sp), Trabecular number (Tb. N), and Trabecular thickness (Tb. Th) were all examined for determining bone histomorphometry in every group. The data demonstrated that bone indicators displayed by Treatment group AIL were significantly greater compared to the animals in the normal saline treatment group, while the 15 mg/kg AIL treatment group demonstrated a superior therapeutic effect (Figures 7B–F). These results showed that the tumor progression of bone metastasis model mice was alleviated when treated with AIL the next day.

Then, HE staining was carried out on the histological sections and the results have been presented in Figure 7A. In the normal saline treatment group, the tibial trabecular bone of nude mice was completely absorbed, the bone cortex was also severely damaged, and the tumor tissue and muscle tissue were severely adhered. In treatment group AIL, bone cortex of nude mice was relatively complete, the bone trabecular structure was visible, and the growth of tumor cells was limited. Compared with the low-dose treatment group, the bone trabeculae of the high-dose treatment group were more closely distributed.

Three-color IF staining results indicated that compared to the animals in the normal saline group, the no. Of TRAP-positive osteoclasts and area of osteoclasts in the drug-treated mice were significantly reduced (Figures 7A, G), indicating that the osteoclasts in the drug-treated group were inhibited. Simultaneously, IF technique was employed to assess the RANKL and IL-1β expression levels in tissues. The results revealed that in comparison to the normal saline group, the RANKL and IL-1β expression in tumor tissues of nude mice in treatment group AIL decreased significantly, with statistically significant differences (Figures 7A, H–I). Immunohistochemical detection of FOXP3 protein in tumor tissue showed that its expression was significantly increased (Figures 7A, J). These analyses suggest that AIL can effectively inhibit the RANKL and IL-1β expression in BC bone metastasis tissues by upregulating FOXP3 in a concentration-dependent manner, and ultimately inhibit the osteoclast differentiation *in vivo* and the progression of the BC bone metastasis.

## Discussion

**FIGURE 8:**
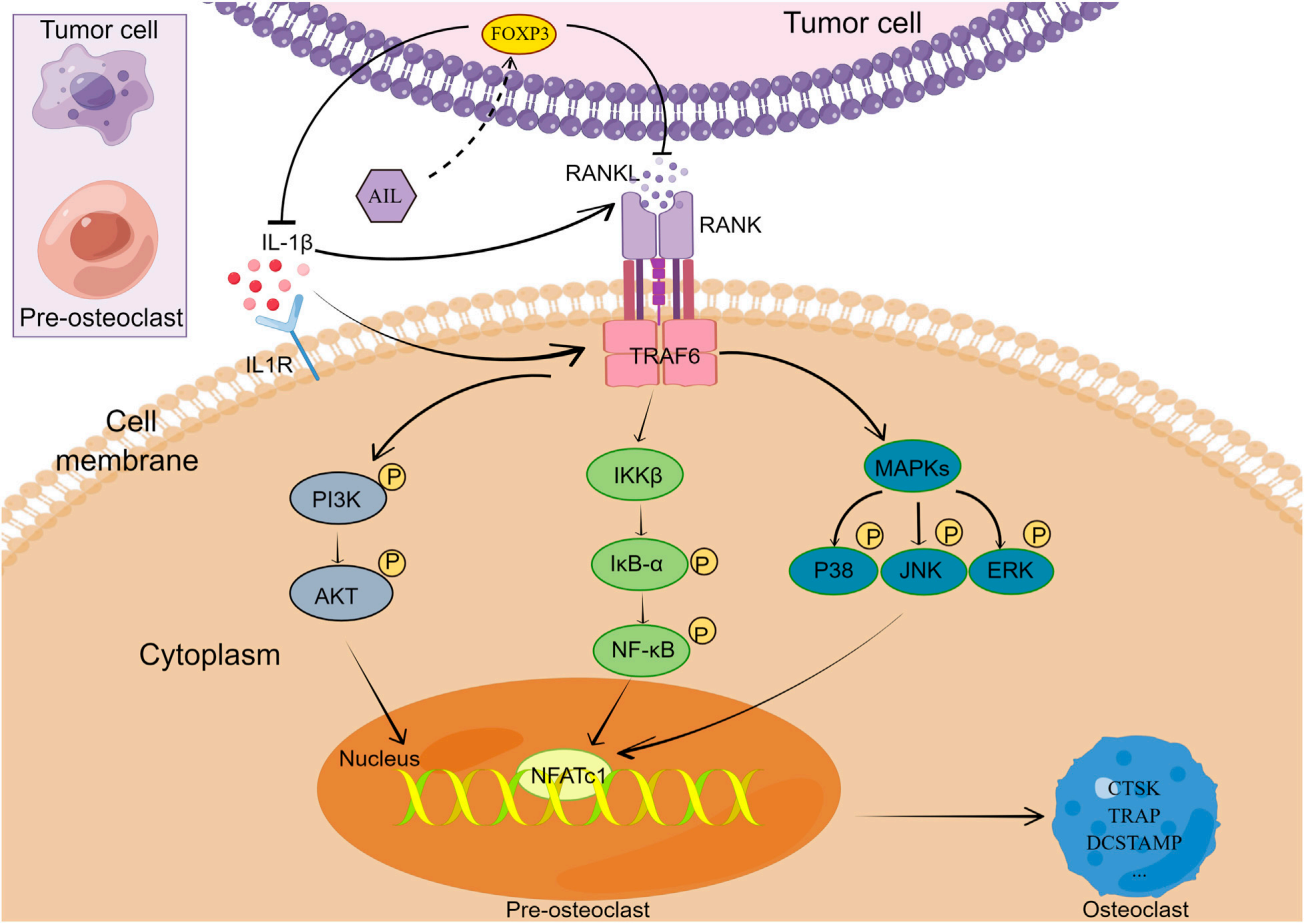
AIL inhibits bone metastasis of breast cancer in multiple ways.

The findings of the *in vitro* study implied that the exposure of MDA-MB-231 and BMM cells to >1.25 μM concentrations of AIL will cause cytotoxicity. Thus, it could be concluded that higher concentrations of AIL may either cause or facilitate the death of the BMM or MDA-MB-231 cells. Therefore, it was anticipated that the concentrations of AIL used in the study (ranging between 0 and 1.25 μM) would not have any deleterious effects on subsequent experiments. The results indicated that AIL can directly prevent the cell proliferation, migration, and infiltration of BC cells or inhibit the expression levels of proteins or genes involved in osteoclast formation, hence preventing bone metastases of BC. A few studies have stated that BC cells secrete soluble compounds in the bone microenvironment that act on osteoclast precursors, and promote the growth of mature osteoclasts, thereby degrading the bones (Shemanko et al., 2016). The findings of the study revealed that AIL inhibits osteoclast formation and maturation by preventing BC cells from secreting a soluble component that regulates bone development. Additionally, Among the cell lines commonly incorporated into xenograft models, estrogen receptor-positive cell lines will only form tumors in the presence of estrogen, and cell lines of the HER2 subtype all have poor tumorigenic ability (Holliday and Speirs, 2011). MDA-MB-231 cells are the best characterized and most reliable cell model for *in vivo* studies of osteolytic bone resorption induced by human breast cancer (Pore et al., 2018). Therefore, the impact of AIL on BC-related bone metastasis was confirmed by constructing a mouse model with MDA-MB-231 to replicate the entire process. In terms of mechanism, AIL inhibits the RANKL and IL-1β secretion in the CM or the BC cells by upregulating FOXP3 in BC cells and also prevents the differentiation and functioning of the osteoclasts induced by the BC cell supernatant by inhibiting the NF-κB, PI3K/AKT, and MAPK signaling pathways (Figure 8).

At present, the research on the pathological process of BC bone metastasis has enabled the development and application of two bone improvement drugs for bone metastasis, namely the third-generation bisphosphonate and humanized anti-RANKL antibody (Maruthanila et al., 2017). Anti-resorption medications are regarded as the existing standard treatment for lowering SRE in bone metastases patients, even though they have altered the course of treatment, improved patient outcomes, and decreased the frequency of bone complications (Liverani et al., 2014). They do, however, have a variety of adverse effects, including hypocalcemia and an imbalance in the homeostasis of calcium. Furthermore, 30%–50% of patients undergoing these treatments still displayed many bone metastases and bone complications (Futakuchi et al., 2016). Additionally, many clinical trials indicated that the bone-modifying medications did not significantly extend the OS duration of the patients during the complete study period (Rordorf et al., 2014). The interaction between the host cells, tumor cells, and the skeletal environment is particularly important for developing osteolytic bone lesions in BC, which creates a pathological vicious cycle. All these cellular interactions are involved in promoting the growth of osteoclasts, which results in aberrant bone tissue degradation and aggressive tumor growth (Patel et al., 2011). Therefore, the main goal of bone metastasis treatment is to break this vicious circle, to minimize bone complications.

Under both normal and pathological circumstances, osteoclasts refer to the host cells that regulate bone resorption. The interaction of RANK and RANKL, which is the primary receptor of RANK ligand, is essential for osteoclast development and maintains the physiological equilibrium through OPG secretion (Hadjidakis and Androulakis, 2006). In addition to increasing the concentration of RANKL and OPG in osteoblasts and driving osteoclasts to progress toward matrix absorption, BC can directly produce soluble media that causes the maturation of osteoclasts. Unbalanced osteoclast activation causes significant bone absorption, which, in turn, encourages tumor growth and increases osteoclast activity (Chen et al., 2010). This feedforward loop develops a favorable milieu in the bone for tumor growth, resulting in bone loss and uncurable cancers. The interactions between the bone microenvironment and the malignant cells can be studied to identify the important pathways and develop targeted treatment strategies. This is the first study that showed that AIL can inhibit the BC-induced osteolytic bone metastasis by reducing the stimulation of breast cancer on osteoclasts and directly targeting BC cells.

Bioactive compounds from natural resources have become a milestone in the treatment of various tumors, which is particularly important for developing drugs for various tumor treatments (Goyal et al., 2017). AIL showed a significant *in vitro* anti-cancer effect on different cancer cell lines, while the malignant cell lines like hepatocellular carcinoma, non-small cell lung cancer, and castration-resistant prostate cancer, showed an effective *in vivo* anti-tumor activity (Bailly, 2020). This study showed that AIL inhibits the *in vitro* proliferation, migration, and the infiltration of BC cells. However, it was unclear whether AIL could be employed for treating the BC bone metastases. Therefore, this study was implemented to evaluate the influence of AIL on the bone metastases of BC and determine its probable molecular mechanisms. AIL significantly decreased the no. Of activated osteoclasts surrounding the bone trabecula and reduced the bone degradation in mice with BC bone metastases, as demonstrated by the *in vivo* HE staining, immunohistochemistry, immunofluorescence staining, *in vivo* imaging, and μCT. The findings of the F-actin ring formation, TRAP staining, and the bone resorption assay showed that AIL significantly decreased the osteoclast cell differentiation and functioning in BMM cells that were stimulated by RANKL and MDA-MB-231CM *in vitro*. Thus, it was concluded that AIL could be used as the probable drug molecule for treating BC and the resulting bone metastases.

Hematopoietic stem cells give rise to osteoclasts, and M-CSF and RANKL two essential chemicals that also play a role in osteoclast function and survival are essential for osteoclast development (Edwards and Mundy, 2011). A previous study and this study also confirmed that RANKL and M-CSF can induce BMM cells to differentiate into osteoclasts. One study found that CXCR4, MMP1, IL-11, and CTGF genes were highly expressed by transcriptome sequencing of bone metastatic MDA-MB-231 cells. These overexpression genes mainly encode cell surface and secreted proteins, and each protein has the function of changing the host tissue environment to promote the formation of osteolytic bone lesions (Kang et al., 2003). A cell adhesion molecule called CDH11, which is overexpressed in human invasive breast carcinoma tissue compared to normal breast tissue, is expressed in the bone microenvironment (Pohlodek et al., 2016). The BC cells that are attracted to the bone, express the Runx2 gene, which then upregulates the VEGF and MMP-9 expression to stimulate the *in vitro* migration and infiltration of the BC cells (Pratap et al., 2005). TGF-β stimulates the migration and infiltration of the MDA-MB-231 BC cell line (Ye et al., 2013; Derynck et al., 2021). In this study, the results indicated that the same genes and proteins were over-expressed in MDA-MB-231 cell lines, and could be inhibited by AIL. It was noted that AIL inhibited the growth, migration, and infiltration of the BC cells, and also suppressed the osteolytic bone damage caused by the BC cells.

Earlier studies have shown that the generation of osteoclasts is regulated by many factors. BC cells stimulate production through indirect or direct RANKL secretion (Owen et al., 2013). RANK is expressed on osteoclast precursors derived from bone marrow, and RANKL/RANK signal pathway may be the main pathway involved in osteoclast formation (Udagawa et al., 2021). An earlier study showed that 50 ng/ml RANKL can induce osteoclast formation. Under other conditions unchanged, this study reduced the amount of RANKL induction to 50%, and added MDA-MB-231 CM. According to the results noted in the study, the no. Of simultaneously-activated osteoclasts and RANKL concentration of 50 ng/ml showed no statistically significant difference. This implies that MDA-MB-231CM induces osteoclast differentiation. A different study has shown that the CM of the BC cells recruits the osteoclast precursors, thereby increasing the quantity and activity of the osteoclasts in mice (Yue et al., 2022). It has been indicated that the interaction between BC cells and bone microenvironment is involved in the complex molecular mechanisms involved in breast cancer bone metastasis. BC cells secrete several key osteolytic factors, including IL-1, IL-6, PTHrP, and TGFβ, which can, either directly or indirectly, promote osteoclast differentiation and functioning (Le Pape et al., 2016). This study came to the same conclusion, and it was noted that the BC-induced MDA-MB-231 CM cells showed an overexpression of the RANKL and IL-1β cytokines. The activation of osteoclasts by several proinflammatory cytokines, like TNFα and IL-1, results in bone degradation (Li et al., 2000). An earlier study revealed that TNFα activates a different pathway in addition to RANKL for activating TRAP and CTSK, which promoted the production of osteoclasts (Li et al., 2000). In addition to mediating TNFα expression by directly promoting osteoclast precursor differentiation and stimulating the RANKL overexpression in stromal cells, IL-1 also plays a synergistic function in RANKL-stimulated osteoclast genesis (Wei et al., 2005). BC cells release an osteolytic factor that promotes the RANKL/RANK pathway, which in turn increases osteoclast activity (Martin and Johnson, 2021). Increased bone resorption leads to a positive feedback loop. Bone matrix releases growth factors like TGF-β into the metastatic microenvironment, further stimulating the proliferation of tumor cells, thus producing more osteolytic factors (Rao et al., 2018). A recent study also shows that breast cancer cells are more metastatic and invasive in the bone microenvironment (Zhang et al., 2021). Therefore, blocking this vicious circle provides a promising field for treating bone metastasis and maintaining the stability of the bone microenvironment. The findings in this study indicated that the RANKL and IL-1β levels in cell supernatant were significantly decreased after treatment with AIL. It was noted that the inhibitory effects of AIL on BC-related bone metastasis may be partly ascribed to the inhibition of cytokine expression. The specific mode needs further experiments to verify. Inflammatory mechanism in breast cancer affects tumor occurrence and metastasis progress (Grivennikov et al., 2010). It was previously reported that FOXP3 can reduce the secretion of inflammatory factors related to osteoclast formation, such as RANKL (Chen et al., 2020). The PCR, WB, and IHC results in the study also verified that FOXP3 was upregulated in AIL-treated MDA-MB-231 cells.

After RANKL binds to RANK, it transduces signals by recruiting the TNF Receptor-related Factors (TRAFs), like NF-κB, MAPK (P38, JNK, and ERK), or AKT. This activated the transcription factors, like the NF-B, c-Fos, or primary transcription regulator Nuclear Factor of the activated T-cell (NFATc1), which induced the osteoclast gene expression, including TRAP, CTSK, DCSTAMP, Matrix Metalloproteinase-9 (MMP-9), calcitonin receptor, etc. (Lee et al., 2006; Ke et al., 2015). The Western Blotting experiments conducted in this study indicated that the addition of AIL-treated MDA-MB-231 CM inhibited the activation of the MDA-MB-231 CM-induced PI3K and AKT signal transduction pathways as well as the phosphorylation of P38, NK, ERK, P65, IκBα protein activation. It is therefore hypothesized that some cytokines in the MDA-MB-231 CM induce BMM cell differentiation into osteoclasts through many signaling pathways, out of which at least one signaling pathway would be essential to osteoclast function. PCR was also employed for assessing the expression of osteoclast markers stimulated by MDA-MB-231 CM and RANKL. The BMM cells induced by the AIL-treated MDA-MB-231 CM showed a significant decrease in the CTSK, TRAP, NFATc1, DCSTAMP, and MMP9 expression. In conclusion, AIL can inhibit the production of osteoclasts by suppressing the secretion of some cytokines by MDA-MB-231 CM, which, in turn, inhibits the PI3K/AKT, NF-κB, and MAPK signaling pathways. Also, AIL suppressed the expression of genes and proteins linked to osteoclast function that were stimulated by RANKL and MDA-MB-231 CM. These findings suggest that a complex interaction between tumor cells, osteoclast precursor cells, and osteoclasts may be responsible for AIL’s suppression of BC bone metastasis.

Considering many limitations in this study, a comprehensive preclinical investigation is needed to prove and deeply understand the molecular mechanism of these potential molecules showing anti-tumor growth and anti-osteolytic activity, to develop a reliable therapy for BC-induced osteolytic bone diseases. Secondly, the results did not indicate which protein was bound to AIL, for the inhibition process. Subsequently, the method of AIL coupling biotin can be used for immunoprecipitation or mass spectrometry detection to determine its interacting proteins. Finally, if *in vitro* studies can completely simulate the microenvironment of BC-related bone metastasis or explore the interactions between tumor and bone microenvironment in animal experiments, more accurate results may be obtained. The acute toxicity experiments of AIL in mice showed that the digestive system was mainly affected by AIL, with the stomach was identified as the main target organ (Tang et al., 2019). This may limit its development into an anticancer drug, and in subsequent studies it may be possible to reduce its toxic effects by, for example, targeting the drug to specific cells through liposome encapsulation.

In conclusion, this study assessed the effect of AIL on osteoclast differentiation induced by cytokines secreted by BC cells and its possible mechanisms. AIL altered the microenvironment of BC bone metastases by upregulating FOXP3 expression in BC cells, and then inhibited osteoclast formation induced by the BC-CM through NF-κB, MAPK and PI3K/AKT signaling pathways. In a mouse model of BC-induced bone metastases, AIL also reduced tumor-stimulated osteolytic bone resorption. The findings suggested that AIL might be a useful therapeutic strategy for the management of BC-related bone metastases.

## Data Availability

The original contributions presented in the study are included in the article/Supplementary Material, further inquiries can be directed to the corresponding authors.

## Supplementary material

Supplementary Figure S1 AIL inhibits the growth of BC transplant tumors in bone tissue in vivo. **(A)** Modeling bone metastases from tibial breast cancer following therapy with various concentrations of AIL. **(B)** The length of tibial malignant modeling tissue was determined. **(C)** The width of tibial malignant model tissue was determined. **(D)** The tumor tissue’s volume was determined. The mean ± SD were used to express all the data. **p*<0.05, ***p*<0.01, in comparison to the control animals.
